# Complete Genome Assembly of *Enterococcus faecalis* 29212, a Laboratory Reference Strain

**DOI:** 10.1128/genomeA.00968-14

**Published:** 2014-09-25

**Authors:** T. D. Minogue, H. E. Daligault, K. W. Davenport, S. M. Broomall, D. C. Bruce, P. S. Chain, S. R. Coyne, O. Chertkov, T. Freitas, H. S. Gibbons, J. Jaissle, G. I. Koroleva, J. T. Ladner, G. F. Palacios, C. N. Rosenzweig, Y. Xu, S. L. Johnson

**Affiliations:** a Diagnostic Systems Division (DSD), United States Army Medical Research Institute of Infectious Diseases (USAMRIID), Fort Detrick, Maryland, USA; b Los Alamos National Laboratory, Los Alamos, New Mexico, USA; c ECBC, Aberdeen Proving Ground, Maryland, USA; d Center for Genome Sciences (CGS), USAMRIID, Fort Detrick, Maryland, USA

## Abstract

*Enterococcus faecalis* is a nonmotile Gram-positive coccus, found both as a commensal organism in healthy humans and animals and as a causative agent of multiple diseases, in particular endocarditis. We sequenced the genome of *E. faecalis* ATCC 29212, a commonly used reference strain in laboratory studies, to complete “finished” annotated assembly (3 Mb).

## GENOME ANNOUNCEMENT

*Enterococcus faecalis* can be both a healthy human gut community member and the causative agent of endocarditis, bacteremia, and urinary tract and other infections. The species is often resistant to multiple antibiotics, displaying both inherent and acquired traits (1). *E. faecalis* ATCC strain 29212 was isolated from a human urine sample collected in Portland, Oregon, United States, and is a commonly used laboratory strain for research (it has been noted in over 500 publications).

High-quality genomic DNA was extracted from a purified isolate using a QIAgen Genome Tip-500 at USAMRIID-DSD. Specifically, a 100-mL bacterial culture was grown to stationary phase and nucleic acid extracted as per manufacturer’s recommendations. Sequence data included both Illumina and 454 technologies (2, 3). We constructed and sequenced an Illumina “standard” library of 100-bp reads at 300-fold genome-coverage and a separate long insert (8,111 ± 1,087-bp) paired-end library (Roche 454 Titanium). The two libraries were assembled together in Newbler (Roche) and the consensus sequences computationally shredded into 2-kbp overlapping fake reads (shreds). The raw reads were also assembled in Velvet, and those consensus sequences were computationally shredded into 1.5-kbp overlapping shreds (4). Draft data from all platforms were then assembled together with Allpaths and the consensus sequences computationally shredded into 10-kbp overlapping shreds (5). We then integrated the Newbler consensus shreds, Velvet consensus shreds, Allpaths consensus shreds, and a subset of the long-insert read pairs using parallel Phrap (High Performance Software, LLC). Possible mis-assemblies were corrected and some gap closure accomplished with manual editing in Consed (6–8).

Automatic annotation for the *E. faecalis* ATCC 29212 genome utilized an Ergatis based workflow at Los Alamos National Laboratory (LANL) with minor manual curation. The genome consists of one chromosome (2,939,973 bp, 37.5% G+C content) and two plasmids (41,610 bp, 34.3% G+C content, and 66,648 bp, 32.9% G+C content, respectively). Annotation located 2,933 coding sequences, 12 rRNA sequences, and 61 tRNA sequences. All rRNA/tRNA and 96% of the protein coding sequences are located on the chromosome. The annotated genome sequence is publicly available, and the raw data can be provided upon request. Preliminary review of the annotated genome found multiple virulence factors noted by Arias and Murray (1). Further analysis into the intraspecies genetic differences, including this strain, is under way.

### Nucleotide sequence accession numbers.

The NCBI accession numbers for this genome are CP008816 (chromosome) and CP008814 to CP008815 (plasmids).
